# ADVERSE EFFECTS OF INTERNET USE ON SUBJECTIVE SOCIAL STATUS AND DEPRESSION SYMPTOMS AMONG CHINESE OLDER ADULTS

**DOI:** 10.1093/geroni/igad104.1232

**Published:** 2023-12-21

## Abstract

Internet use has been found to be associated with decreases in depressive symptoms among older adults. However, past evidences may have had publication bias due to the negative consequences of internet use demonstrated in other age groups. Thus, this study aims to investigate the adverse effect of older adults’ internet use on depressive symptoms and its mechanism. The inverse subjective social status (SSS) in depressive symptoms is a well-established research finding. Based on this, we hypothesize that older adults’ internet use could expand their reference group and exposing them to more ageist content, thus reducing their SSS which in turn increases depressive symptoms. We examined how older adults’ internet use influences SSS and how SSS mediates the effect of internet use on depressive symptoms in China. The data were from wave 3 (2016, T1), 4 (2018, T2), and 5 (2020, T3) of the China Family Panel Studies, a nationally representative survey. The longitudinal mediation analysis included 3,237 participants who were aged 60 and above at T1 (wave 3) and were followed through waves 4 and 5. As expected, Internet use at T1 predicted lower levels of SSS at T2. Lower levels of SSS at T2 predicted higher levels of depressive symptoms at T3. This medicating effect was significant. This time-varying mediation which appeared to be explained by the relative deprivation and ageism following internet use among older adults. This study theorizes and provides evidence for previously overlooked psychosocial pathway through which internet use among older adults produces negative consequences.

